# Temporal momentum: an online replication and beyond

**DOI:** 10.1007/s00426-025-02167-4

**Published:** 2025-11-05

**Authors:** Manuel Vencato, Marco Zorzi, Mario Bonato

**Affiliations:** 1 Department of General Psychology and Padova Neuroscience Center, University of Padova, Padova, Italy; 2 IRCCS San Camillo Hospital, Venice, Italy

## Abstract

Performing mental arithmetic on brief temporal durations has been recently shown to induce operation-specific distortions. In a time reproduction task, addition resulted in longer responses while subtraction induced shorter responses, despite their identical arithmetic outcome (Bonato et al., *Cognition*, *206, *2021). This effect has been named temporal momentum, in analogy with the representational momentum found when representing the position of moving objects, and it mirrors the operational momentum characterizing mental arithmetic with numerical quantities. In Experiment 1, we assessed the reliability of the temporal momentum effect in the first direct replication of Bonato et al.‘s temporal arithmetic task by using an online procedure for data collection. In Experiment 2, we also tested whether under-estimation in subtraction could be attributed to the longer operand being always presented first in the original study. The results showed a reliable temporal momentum effect that was virtually indistinguishable from previous, laboratory-based experiments. Moreover, in Experiment 2, under-estimation in subtraction was still present when participants had to compute the difference between two operands regardless of their order, thereby excluding that the temporal momentum in subtraction is due to the specific ordering of the stimuli. This pre-registered study further demonstrates that the temporal momentum effect is a robust and reliable marker indexing the mental manipulation of time durations, consistent with the hypothesis that time processing includes some features resembling those involved in spatial processing.

## Introduction

Possibly due to evolutionary pressure, every human is equipped with mechanisms for efficiently representing quantities in the domains of space, time, and numbers. It has been suggested that these domains share several features that indicate a common system for quantity representation (Walsh, 2003), and multiple research lines have indeed described correlations among them (Cantlon et al., 2009; De Hevia et al., 2014; Dehaene & Brannon, 2011; but see Hamamouche & Cordes, 2019, for an alternative hypothesis).

Within this context, systematic processing biases might index commonalities or shared components across these different domains. Our study focuses on the widely reported associations between quantity/duration/order and space (Bonato et al., 2012, for review). This association is consistently observed in tasks requiring the categorization of one feature of the stimuli, such as magnitude or parity for numbers, as well as order or duration for time. These systematic biases are thought to reveal core characteristics, spatial in this case, of the underlying representation(s). Specifically, when responses are performed by using a left-sided effector, they are faster and more accurate for relatively “small”, “short”, or “early” ordered items compared to relatively “large”, “long”, or “late” items, whereas the opposite preference is found for responses performed with a right-sided effector. It has been suggested that the environment plays an important role in establishing these associations, as right-to-left reading cultures show an opposite preference for both numbers (Dehaene et al., 1993) and ordered items (Guida et al., 2018). Nevertheless, the picture is likely more complex as some space-quantity associations (e.g., preference for a pairing between a certain quantity and spatial position) can also emerge in the absence of cultural modulation, as they are observed in chicks (Rugani et al., 2015) and their direction can be flexibly changed by ad-hoc manipulations (Fischer et al., 2010; see also Ranzini et al., 2016).

In short, mounting evidence supports the interaction between several, heterogenous factors in determining the association between time/number and space. Despite some methodological criticisms, these associations have been mostly investigated using lateralized responses (not only in humans but also in animals). Many scholars believe that these effects represent the behavioral signature of a spatial representation that typically aligns with the direction of commonly performed movements or shifts of attention, such as reading and writing (see Bonato et al., 2012 for a review). These associations are reliably found in healthy participants (Bonato et al., 2012; Vallesi et al., 2008) as well as in neurological patients. In the presence of spatial disorders due, for instance, to unilateral brain damage, these associations become asymmetric, as if they were resembling contralesional spatial impairments characterizing the neuropsychological syndrome called spatial neglect. Individuals with right brain damage and left neglect may display selective distortions for “early” or “short” elements/durations, resembling the deficits observed in contralesional space processing (Bonato et al., 2016; Marin et al., 2016; Saj et al., 2014; Zorzi et al., 2002, 2012).

The widespread detection of space-time associations, encompassing both temporal durations and temporally-ordered events (see Previtali et al., 2010; van Dijck & Fias, 2011), indicates a surprising similarity in the mental representation of two very different aspects of time, namely sensory and conceptual time (Bonato & Umiltà, 2014). While this similarity might suggest the presence of supramodal mechanisms supporting the processing of magnitudes and durations, it has been argued that the ubiquity of these associations might result from the response layout (Fischer et al., 2010). As a consequence, a common criticism of these associations is that spatial aspects may not be genuinely and spontaneously present but rather induced, for instance, by the use of binary, lateralized, responses (Proctor & Cho, 2006).

A recent study by Bonato et al. (2021) revealed a new marker of the time-space interactions in a task not requiring any lateralized response and therefore not subject to the previously mentioned putative induction of a spatial association (for a discussion of this issue see Bonato et al., 2016; van Dijck & Fias, 2011). The effect did not emerge while processing single durations but, rather, it was a consequence of adding or subtracting durations, that is, performing temporal arithmetic. Participants were asked to add or subtract the duration of pairs of sounds in a time reproduction task, using pairs that would yield identical target duration (e.g., 750ms) for addition (e.g., 600ms + 150ms) and subtraction (e.g., 1200ms − 450ms). The reproduced durations were significantly longer for addition trials compared to subtraction trials. This newly discovered time distortion, named “*temporal momentum*”, was considerably large in size (20–40% error, depending on the duration) and it remained present after controlling for the potential bias induced by the mere presence of the operation sign (+/-) and for the baseline error in reproducing each target duration without performing arithmetic operations.

The “*temporal momentum*” effect revealed by Bonato et al. (2021) seems to be the counterpart of the *“operational momentum”* effect in the number domain, whereby a pattern of overestimation for addition and underestimation for subtraction was first shown for operations on (non-symbolic) visual numerosities (McCrink et al., 2007) and then extended to Arabic digits (Knops et al., 2009). The term “operational momentum” was coined after a well-known perceptual bias known as “representational momentum”, which refers to the finding that the position of a moving objects is misperceived along its trajectory (see Hubbard, 2017) for review). Finding a conceptually similar effect in the temporal domain seems therefore suggestive of a tight coupling with space for both time and numbers. As argued by Hubbard (2017), momentum-like effects are the behavioral signature of a supramodal mechanism shared among different domains which implements the processing of movement in space. This framework allows interpreting the temporal momentum findings as possibly due to an attentional shift along a mental timeline spatially characterizing time flow (Bonato et al., 2012; see also Guida et al., 2018b). This mirrors the representational momentum in the numerical domain, which is also interpreted as due to an operation-specific direction/attentional shift (Knops et al., 2014; McCrink et al., 2007). Nevertheless, further investigation of the temporal momentum effect is crucial to substantiate the attentional interpretation. Notably, another empirical effect suggests an association between spatial attention and arithmetic in the form of lateralized shifts of attention being modulated by the type of arithmetic operation to be performed (Masson & Pesenti, 2024, 2016). This space-arithmetic association has been compared with the operational momentum in few studies (for review see Haman & Lipowska, 2024; Prado & Knops, 2024). Results however were not conclusive in finding a common origin for the two effects. For the case of temporal momentum, any comparison with other effects attributed to spatial processing remains, for the moment, only an hypothesis. Our initial findings still await replication and it is possible that the shorter durations found for subtraction problems are simply due to the stimuli characteristics. For instance, the decreasing order of the operands might have triggered an anchoring effect with respect to the (shortest) second operand.

The aim of the present study was to further extend the investigation of the temporal momentum effect in two experiments performed online. As in Bonato et al. (2021), participants had to add, subtract, or merely reproduce the temporal duration of auditory stimuli (white noise). Experiment 1 was a direct replication of the original laboratory-based study by Bonato et al. (2021, Experiment 2). The online replication was also meant to test the reliability of the effect against the variability introduced by participant-specific testing equipment and environment. The high variability in the audio hardware devices together with the potential environmental noise/heterogeneity might be a challenge towards successful replication of the original findings. Experiment 2 aimed at better understanding the conditions triggering the temporal momentum effect and more specifically it examined the effect of operand duration on temporal arithmetic. In our previous study there was a clear modulation due to the duration of the second operand, suggesting that the temporal momentum effect is influenced by perceptual anchoring (LeBoeuf & Shafir, 2006). Indeed, regression modeling demonstrated that both the type of operation and second operand duration were significant predictors of the temporal estimates. Moreover, we addressed the potential role of increasing vs. decreasing durations. This effect has been for instance described in an experiment pairing line drawing with arithmetic (Shaki et al., 2015), whereby participants drew a longer line when paired with a sum with increasing vs. decreasing operands. In Bonato et al. (2021) the possibility that operand order plays a role for subtraction problems could not be fully disentangled by the effect of operand duration because all subtraction problems were characterized by a second operand shorter than the first operand. As a consequence, participants were experiencing two stimuli decreasing in duration while response (outcome of the subtraction) had to be always longer (up to three or four times) than the second operand. The momentum effect for subtractions (outcome durations shorter than expected) could therefore have been due to anchoring effects, which are not restricted to conceptual estimates but can affect, for instance, visual proportions and supra second time durations (Thomas & Handley, 2008).

## Methods

The study was approved by the University of Padova Ethics Committee for Psychological Research, and it was conducted in agreement with the Declaration of Helsinki guidelines. Data collection occurred online for both experiments. Participants were contacted by the first author and received the link for participating in each experiment via e-mail. They all gave their informed consent before participating. Both experiments were pre-registered on the Open Science Framework (osf.io/4r2kh).

### Software components

The JsPsych framework (de Leeuw, 2015) was used for the development of the experimental tasks and the Jatos framework (Lange et al., 2015) was used for the upload of the experiment. The “jspsych-psychopshysics” plugin (Kuroki, 2021) allowed to optimize the timing of the stimuli. Data were analyzed with R (R Core Team, 2021; RStudio Version 1.4.1106).

### Participant inclusion and exclusion criteria

Participants were naïve with respect to the purposes of the study. Prerequisites for participation were age between 20 and 30 years and the use of the “Chrome” web browser. We decided three a-priori criteria for excluding a participant in case one of the following conditions were met: (i) more than 15% of discarded trials; (ii) overall duration of the experiment above 90 min or below 15 min; (iii) exiting the “Fullscreen mode” more than 5 times. Trials were discarded for each participant when responses fell two SDs outside the individual average for each condition and target duration, as well as when responses had onset > 30 s.

## Experiment 1

### Methods Experiment 1

#### Sample size and number of trials

32 participants (average age 25.2 years, SD 2.42) took part in Experiment 1. No participants had to be removed according to the previously described criteria. Each participant performed 144 trials, divided into 9 blocks (3 blocks for each operation condition). Each operation Condition (addition, subtraction, baseline (no operation/reproduction) included 4 Target Durations (600ms vs. 750ms vs. 900ms vs. 1050ms). There were four combinations for each temporal duration (see Table 1). Addition and subtraction problems were presented in alternated blocks, order was counterbalanced across participants. The sample size and the number of trials was calculated according to the findings by Bonato et al. (2021, Experiment 2) by using the “power contours” as calculated by an automatic tool (Baker et al., 2020).

**Table 1:** The different temporal durations (in milliseconds) for addition (left), subtraction (center), and baseline (right) condition used in Experiment 1, reported separately for the first and the second operand. The stimuli were the same as in Bonato et al. (2021, Experiment 2)

Addition			Subtraction			Baseline (no operation performed)		
First operand	Second operand	Total	First operand	Second operand	Total	First operand	Second operand	Total
450	150	600	750	150	600	600	/	600
150	450	600	900	300	600	750	/	750
400	200	600	1050	450	600	900	/	900
200	400	600	1200	600	600	1050	/	1050
150	600	750	900	150	750			
600	150	750	1050	300	750			
300	450	750	1200	450	750			
450	300	750	1350	600	750			
150	750	900	1050	150	900			
750	150	900	1200	300	900			
300	600	900	1500	600	900			
600	300	900	1650	750	900			
300	750	1050	1200	150	1050			
750	300	1050	1350	300	1050			
600	450	1050	1500	450	1050			
450	600	1050	1650	600	1050			

#### Temporal arithmetic task

**Fig. 1:**
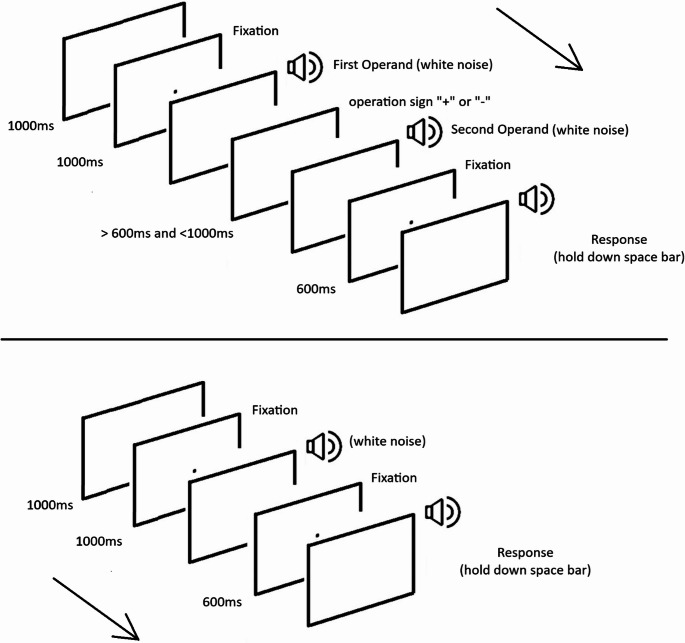
Schematic representation of the temporal arithmetic task (above) and of the baseline (no operation/reproduction only) condition (below). The word “VAI!” (Italian for GO!) was presented at the end of the trial, immediately before response, while in the first screen the Italian for “wait” was presented (not shown here)

The procedure was identical to Experiment 2 in Bonato et al. (2021). Each trial started with the message “Wait…” (1000 ms; in Italian “Aspetta…”) followed by a fixation point (1000 ms) and by an auditory stimulus (first operand). Then, the “+” or “-” symbol appeared for a random time interval between 600 ms and 1000 ms. This random duration was introduced to avoid the use of heuristics based on the total duration (Takahashi & Watanabe, 2015). When the symbol disappeared, the second auditory stimulus (second operand) was played and immediately followed by a fixation point lasting 600 ms. Then the message “GO!” (in Italian “VAI!”) prompted the participant for responding by pressing the space bar for a duration corresponding to the outcome of the operation (i.e., first operand -/+ second operand). During the pressure of the space bar the word “GO!” disappeared and an auditory stimulus was played until the space bar was released (See Fig. 1 for a schematic representation of the task). Participants were informed in advance about the type of block (addition or subtraction or baseline i.e. no operation”). In the subtraction block the first operand was always longer than the second operand to avoid “negative” durations. Each block was preceded by 9 training trials.

#### Baseline task (reproduction only/no operation performed)

After the presentations of the message “Wait…” (1000 ms) and a fixation point (1000 ms) an auditory stimulus (white noise) with different durations was played. After the presentations of a further fixation point (600 ms) a written “GO!” message (VAI!) prompted the participant to press the space bar. When the participant pressed the space bar an auditory stimulus was played and the word “GO!” disappeared (See Fig. 1).

#### Stimuli

The temporal duration of the different white noise stimuli is shown in Table 1. Note that each target duration for “Subtraction” and “Addition” was determined by four different combinations of the two operands. In the “Addition” condition, half of the pairs were characterized by a second operand longer than the first while for half of the pairs it was the opposite. In the “Subtraction” the first operand was always longer, as in Bonato et al. (2021).

### Results Experiment 1

**Fig. 2:**
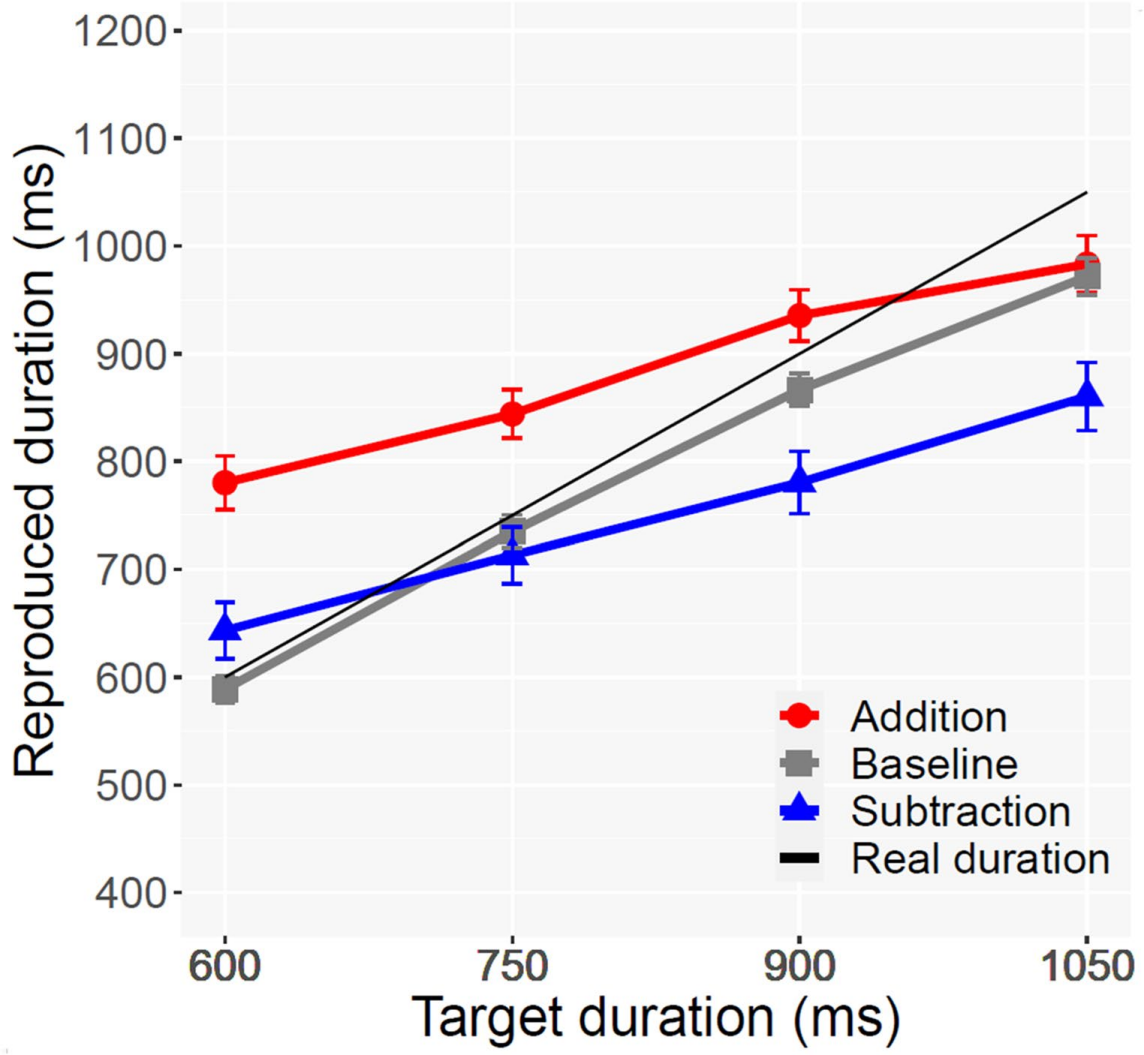
Average duration reproduced for each condition in Experiment 1. In the Baseline condition no operations were performed and participants were asked to press the response key for a duration corresponding to the stimulus presented. Error bars show the standard error of the mean

None of the participants met the pre-registered exclusion criteria. In accordance with the trial exclusion criteria described above, 2.67% of the trials were removed. No response had to be removed due to an onset > 30s. A repeated measures ANOVA with 4 Target Duration conditions (600, 750, 900, 1050) and 3 Operation conditions (Addition vs. Subtraction vs. Baseline) as factors showed significant main effects of Target duration [*F*(1.86,57.66) = 279.7, *p* <.001, *η*_G_^2^ = 0.37, ε = 0.62], and Operation [*F*(2,62) = 21.2, *p* <.001, *η*_G_^2^ = 0.16]], as well as the two-way interaction [*F*(6,186) = 24.5, *p* <.001, *η*_G_^2^ = 0.05]. As can be seen in Fig. 2, despite an identical target outcome duration, the reproduced durations for additions were longer than those for subtractions. Crucially, the longer reproduction for Addition vs. Subtraction was present for all temporal durations, replicating the temporal momentum effect [600 ms: *t*(31) = 5.1, *p* <.001; 750 ms: *t*(31) = 5.4, *p* <.001; 900 ms: *t*(31) = 6.0, *p* <.001; 1050 ms: *t*(31) = 4.83, *p* <.001; Bonferroni corrected]. On the contrary, the difference between the Subtraction and the Baseline condition was significant only for the two longer durations [600 ms: *t*(31) = 2.5, *p* >.05; 750 ms: *t*(31) = 1.16, *p* >.05; 900 ms: *t*(31) = 3.0, *p* <.01; 1050 ms: *t*(31) = 4.2, *p* <.001; Bonferroni corrected]. The Experiment did not include trials without second operands to control for the effect of the operand sign. However, we used the previous results of Bonato et al. (2021) to examine the potential impact of the operand. The differences found between “Addition” and “Subtraction” were still significant across all target durations after correcting the data for the mean bias (39 ms) found by Bonato et al. (2021, Exp. 1) in relation to the mere presence of the “+” operation sign [600 ms: *t*(31) = 3.7, *p* <.001; 750 ms: *t*(31) = 3.8, *p* <.001; 900 ms: *t*(31) = 4.5, *p* <.001; 1050 ms: *t*(31) = 3.3, *p* <.001; Bonferroni corrected] (Fig. 2).

#### Operand effect

A specific analysis tested whether the participants’ estimates in the temporal arithmetic task were also influenced by the specific combination of operands. This analysis was only possible for the addition condition, as in subtraction the first operand was always longer than the second operand (Experiment 2 was specifically designed to address this limitation). ANOVA test for repeated measures with Target Duration and Operand Order (long-short vs. short-long) as factors showed a significant main effect of Target Duration [*F*(1.71, 51.87) = 94.5, *p* <.001, *η*^2^ = 0.23, gg = 0.75]. Similarly, the main effect of Operand Order was statistically significant [*F*(1,31) = 23.3, *p* <.001, *η*^2^ = 0.03] as the Short-Long order (914 ms) determined a larger overestimation than the Long-Short order (857 ms). The two-way interaction was not significant [*F*(3,93) = 2.5, *p* >.05, ns]. As discussed by Bonato et al. (2021), the influence of operand order could be attributed to an anchoring effect (LeBoeuf & Shafir, 2006; Oppenheimer et al., 2008), as participants might have been biased by the last stimulus heard and/or by the order of the operands. It should be noted that, despite this difference, both combinations were characterized by overestimation. Indeed, even additions with Long-Short order (e.g. those resulting in shorter estimates: 857 ms on average) were in any case estimated as significantly longer than both baseline (790 ms; *t*(31) = 3.32, *p* <.01) and subtraction (749 ms; *t*(31) = 4.47, *p* <.001, Bonferroni-corrected).

#### Comparison with previous data

At visual inspection the pattern found in online Experiment 1 seems virtually identical to the findings of Experiment 2 by Bonato et al. (2021), obtained with the same procedure yet performed in a laboratory setting. To compare the two datasets we carried out an omnibus repeated measures ANOVA which included the previously described within participants factors along with Experiment as a between participants factor. The significant within-participants effects were identical to those reported for Experiment 1 alone (i.e., Target Duration, Operation, and their interaction). In contrast, the main effect of Experiment was not significant (*F*(1,54) = 0.55, *p* >.05, ns. Moreover, all the interactions including Experiment were not significant (all ps > 0.2). According to this ANOVA the two datasets (collected in presence vs. online) do not differ.

#### Regression modeling

**Fig. 3:**
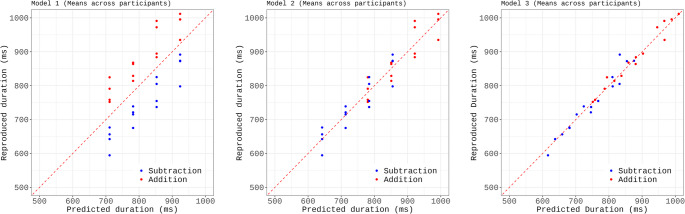
Experiment 1: Scatter plot comparing the observed data (reproduced duration in ms) with the data predicted by the model (Predicted duration), which was fit incrementally using Target Duration (model 1, left panel), Operation Type (model 2, middle panel), and Second Operand Duration (model 3, right panel) as predictors. The alignment of the points along the diagonal line is an index of fitness

We used regression modelling to incrementally build a statistical model that explains the role of the different variables in predicting the reproduced duration for each operation (i.e., item). While Bonato et al. (2021) fitted the mean duration across participants, here we used linear mixed modeling (R package lmer, Maximum-Likelihood estimation) to fit the individual data, including subject as a random effect. Model selection proceeded in a stepwise manner, starting from a null model with only a random intercept for subject. We sequentially added fixed effects and compared nested models using likelihood ratio tests (LRTs). Scatter plots (Fig. 3) contrast the predictions of each model with the empirical data for each of the 32 operations, plotting item means (both observed and predicted) averaged across participants. The inclusion of Target duration improved the fit (Model 1 vs. Model 0, LRT: χ²(1) = 258.71, *p* <.001) but the predictions markedly deviated from the observed data, highlighting the systematic overestimation for addition and underestimation for subtraction (Fig. 3, left panel). Indeed, introducing Operation type (coded categorically as + 1 for addition and − 1 for subtraction) yielded a large improvement of model fit (Model 2 vs. Model 1: χ²(1) = 252.64, *p* <.001; see Fig. 3, middle panel). Finally, we entered the Second operand duration, which further improved the fit (Model 3 vs. Model 2, LRT: χ²(1) = 45.14, *p* <.001) and visibly reduced the prediction error (see Fig. 3, right panel). Therefore, Model 3 was retained as the best-fitting model (see Table 2 for model coefficients). The model accounted for 62% of the variance in the data (conditional R²), with fixed effects explaining 28% of the variance (marginal R²). The Shapiro-Wilk test for normality of residuals was statistically significant (W = 0.997, p =.033), suggesting a slight departure from normality. However, deviation was minimal, and visual inspection of the residuals did not reveal any major concerns. Variance inflation factors were low (all VIFs < 1.1), indicating no issues with multicollinearity.

### Discussion Experiment 1

Experiment 1 closely replicated the outcome by Bonato et al. (2021). Mentally manipulating two temporal durations led to produce longer intervals after addition than after subtraction. Finding results which perfectly overlapped with our previous study was not obvious considering the online setting, the different software and the higher variability due to multiple hardware and testing environments. In the “Addition” condition the overestimation increased when the second operand was longer than the first one. However, even when the first operand of an addition was longer, the overestimation, although smaller, was still detected. This suggests that, for addition, the anchoring effect cannot explain the temporal momentum effect. In contrast, no conclusion about the anchoring effect could be drawn for subtraction problems, as they were characterized by second operands which were always longer than first operands. Regression modeling based on linear mixed models showed that the duration of the second operand was a significant predictor of the arithmetic estimates. This suggests that the categorical effect of operand order observed in the ANOVA might be possibly induced by the duration of the second operand (e.g. the apparent effect of operand order would in fact be a consequence of second operand duration). In Experiment 2, we employed a new set of stimuli that introduced a balanced order of presentation also for the “Subtraction” condition, with the aim to replicate the operational momentum effect and disentangle the potential effects of operand order and second operand duration.

**Table 2:**
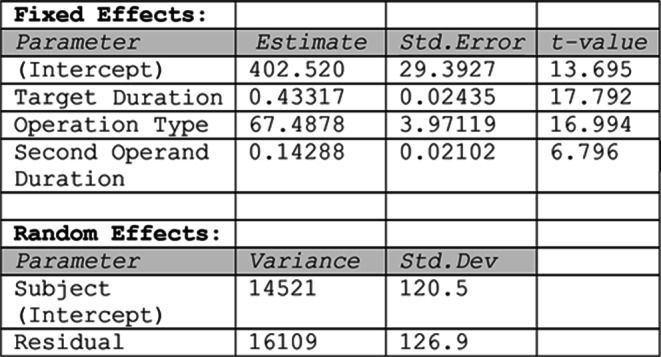
Experiment 1: Summary of the best-fitting Linear Mixed-Effects Model predicting reproduced duration in the temporal arithmetic task. The table shows estimates, standard errors, and t-values for fixed effects, and variance and standard deviation for random effects

## Experiment 2

### Method Experiment 2

32 participants took part in Experiment 2 (average age 25 years, SD 4.49).

#### Temporal arithmetic task

Experiment 2 was run online, using the same software and methods of Experiment 1. The crucial difference with respect to Experiment 1 was that in half of the “Subtraction” trials the first operand was longer than the second while for the other half of trials it was shorter (in Experiment 1 all subtractions problems had a longer first operand). Accordingly, the task instructions were modified in Experiment 2 and participants were asked to calculate a sum/difference between the operands without any explicit reference to their order (as opposed to Experiment 1) “Hold down the space bar until the duration matches the SUM/DIFFERENCE of the two previous sounds”. As in Experiment 1 each block had one type of operation only and participants knew in advance the operation to be performed. The operation sign, acting as reminder, was presented after the second operand. All other task characteristics were identical to Experiment 1.

#### Baseline task (reproduction only/no operation performed)

The Baseline task (no operation performed) was implemented exactly as in Experiment 1.

#### Stimuli Experiment 2

The durations of the sounds used in Experiment 2 are shown in Table 3. As anticipated, as opposed to Experiment 1 for the “Subtraction” condition half of the trials had a longer first operand. The new operands for subtraction problems were determined by minimizing changes as much as possible compared to Experiment 1. For the subtraction block, the second operand was in half of the trials longer and in the other half shorter than the result. By doing so the average anchoring effect (due to second stimulus duration) should be tending to zero. This change is also relevant with reference to the first operand. With the new set of stimuli half of the subtraction trials called for a long keypress after a short first operand vs. long second operand and half of the trials prompted for the opposite. This resembles the characteristics of addition stimuli already present in Experiment 1.

**Table 3:** Experiment 2: The different temporal durations (in milliseconds) for addition (left), subtraction (center), and baseline (right) condition, reported separately for the first and the second operand

Addition			Subtraction			Baseline (no operation performed)		
First operand	Second operand	Total	First operand	Second operand	Total	First operand	Second operand	Total
450	150	600	750	150	600	600	/	600
150	450	600	150	750	600	750	/	750
400	200	600	1050	450	600	900	/	900
200	400	600	450	1050	600	1050	/	1050
150	600	750	1050	300	750			
600	150	750	300	1050	750			
300	450	750	1200	450	750			
450	300	750	450	1200	750			
150	750	900	1200	300	900			
750	150	900	300	1200	900			
300	600	900	1500	600	900			
600	300	900	600	1500	900			
300	750	1050	1500	450	1050			
750	300	1050	450	1500	1050			
600	450	1050	1650	600	1050			
450	600	1050	600	1650	1050			

### Results Experiment 2

While no participant met the pre-registered exclusion criteria, one participant had to be removed from analyses because the average response time (across all conditions) was more than 3 standard deviations higher than the participants’ group mean, causing a marked deviation from normality in the residuals of the mixed linear model analysis. Note, however, that the pattern of results did not change following this exclusion. Additionally, in accordance with the trial exclusion criteria described for Experiment 1, 3.29% of the trials were removed.

**Fig. 4:**
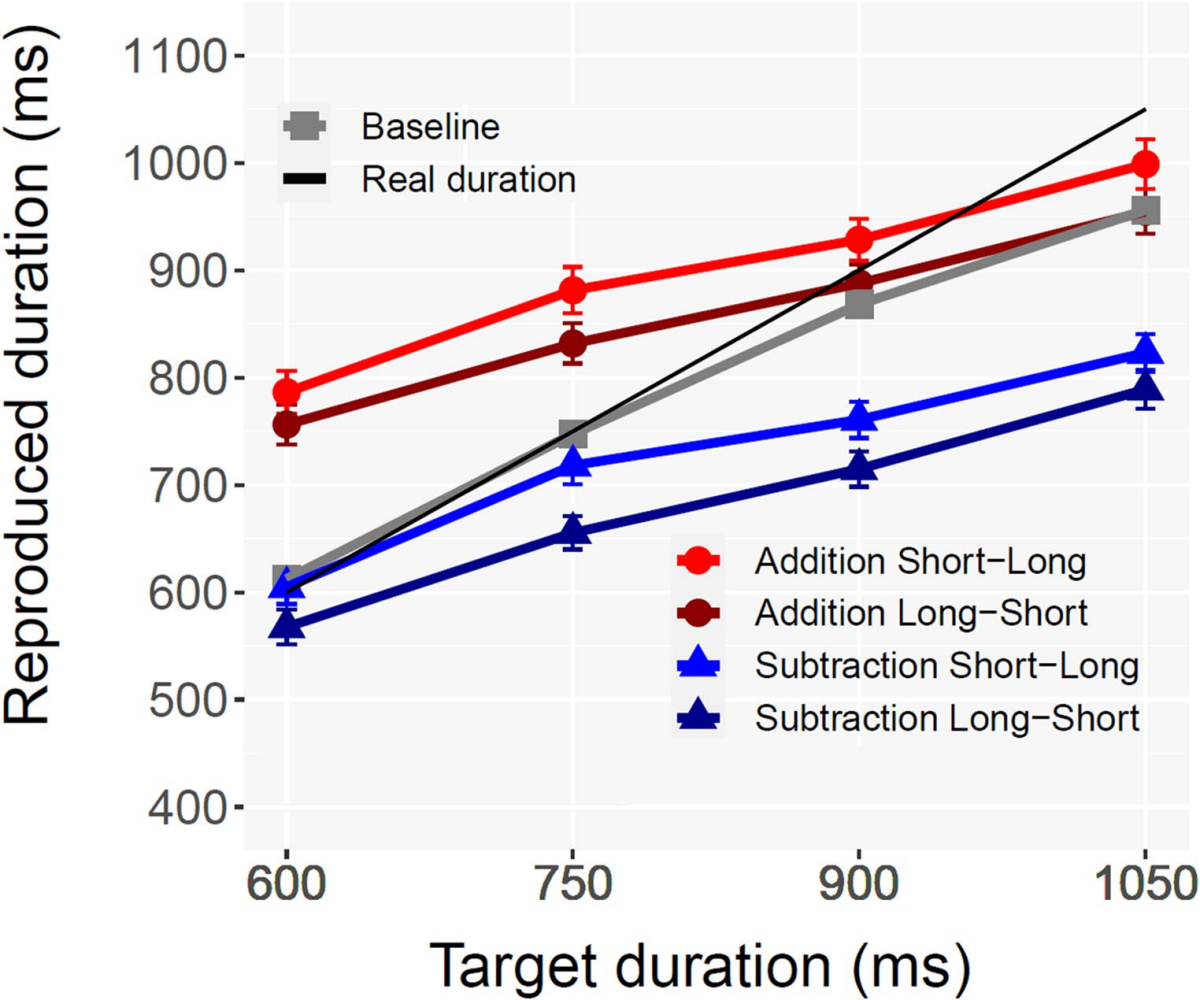
Experiment 2: Average duration reproduced for each condition, reported separately depending on the order of the operands. Error bars show the standard error of the mean

As in Experiment 1, we first carried out a repeated measures ANOVA with 4 Target Duration conditions (600, 750, 900, 1050) and 3 Operation conditions (addition vs. subtraction vs. baseline). We found significant main effects of Target Duration [*F*(1.83,54.9) = 224.03, *p* <.001, ηG2 = 0.30, *ε* = 0.61] and Operation [*F*(1.64,49.2) = 16.01, *p* <.001, ηG2 = 0.19, *ε* = 0.82]. Also the two-way interaction was significant [*F*(4.32, 129.6) = 13.97, *p* <.001, ηG2 = 0.03, *ε* = 0.72]. Both operations significantly differed from baseline: Addition *t*(30) = 2.62, *p* <.05 and Subtraction *t*(30) = 3.91, *p* <.01 (Bonferroni corrected). The mean estimate for Addition (877 ms) was significantly longer than for Subtraction (704 ms) *p* <.001). The difference for Addition vs. Subtraction was present across all target temporal durations [600 ms: *t*(30) = 5.2, *p* < 001; 750 ms: *t*(30) = 4.4, *p* <.001; 900 ms: *t*(30) = 4.6, *p* <.001; 1050 ms: *t*(30) = 4.1, *p* <.01; with Bonferroni correction] and rather constant (see Fig. 4), indexing the presence of temporal momentum for all conditions. As done for Experiment 1, we determined based on Experiment 1 by Bonato et al. (2021) what the impact of the operand sign could have been. The differences found between “Addition” and “Subtraction” were still significant across all durations even after correcting the data for the average bias (38.6 ms) found by Bonato et al. (2021) [600 ms: *t*(30) = 4.11, *p* < 001; 750 ms: *t*(30) = 3.41, *p* <.01; 900 ms: t(30) = 3.52, *p* <.01; 1050 ms: *t*(30) = 3.19, *p* <.05; with Bonferroni correction].

#### Operand effect

We then assessed whether the characteristics of the operands influenced the temporal estimates. We included not only Addition (as already done in Experiment 1) but also Subtraction. This was possible due to the presence of shorter first operands (see Fig. 4). A repeated measures ANOVA (with Target Duration, Operation and Operand Order as factors) showed a significant main effect of Target Duration [*F*(2.25, 67.5) = 121, *p* <.001, *η*G2 = 0.16, ε = 0.75] along with the main effects of Operation [*F*(1,30) = 23.43, *p* <.001, *η*G2 = 0.19] and of Operand Order [*F*(1,30) = 24.41, *p* <.001, *η*G2 = 0.01]. No interaction turned out to be statistically significant. For Addition, the “Short-Long” order (896 ms) triggered answers lasting longer than the “Long-Short” order (858 ms) *t*(30) = 3.55, *p* <. 01. Similarly, for Subtraction the “Short-Long” order (726 ms) triggered significantly longer answers than the “Long-Short” order (682 ms) *t*(30) = 3.73, *p* <.001. As mentioned for Experiment 1 this effect could be due to anchoring related to the Second Operand Duration (LeBoeuf & Shafir, 2006) or to the experienced increasing/decreasing of the two operands. Thanks to the presence of addition and subtraction problems with This issue was disentangled using regression modeling (see below). The difference between addition problems and subtraction problems was significant even considering only those subtraction problems with a longer second operand and only those addition problems with a shorter second operand [*t*(30) = 3.85, *p* <.001].

#### Comparison with previous data

At visual inspection the outcomes of Experiment 1 and Experiment 2 seem almost identical. To statistically test this similarity we carried out an ANOVA with “Experiment” as between-participants factor variable. The significant within-participants effects were identical to those reported for each single experiment (i.e., target duration, operation, and their interaction). In contrast, the main effect of Experiment was not significant (*F*(1,61) = 0.41, *p* >.05) and it did not interact with the other factors.

In the same vein, as for Experiment 1, we did not find a significant main effect or interaction involving Experiment when comparing current Experiment 2 with Experiment 2 in Bonato et al. (2021) (*F*(1,53) = 1.63, *p* >.05).

In short, the pattern of findings was stable regardless of data collection modality and change in the operands (subtraction).

#### Regression modeling

As for Experiment 1 we used linear mixed-effects modeling (R package lmer, Maximum-Likelihood estimation) to analyse the temporal arithmetic data. We also used this approach to disentangle the potential effects of Operand order and Second operand duration. Though predicting an effect in the same direction, Operand order is categorical and implies a fixed-size bias, whereas Second operand duration is continuous and it should induce a variable bias. Model selection proceeded in a stepwise manner, starting from a null model with only a random intercept for subject. We sequentially added fixed effects and compared nested models using likelihood ratio tests (LRTs). The inclusion of Target duration (Model 1 vs. Model 0, LRT: χ²(1) = 183.90, *p* <.001) and Operation type (Model 2 vs. Model 1, LRT: χ²(1) = 284.83, *p* <.001) significantly improved model fit. At step 3, we compared two non-nested models: one including the Second Operand Duration (Model 3a) and one including Operand Order (Model 3b), while retaining the fixed effects from Model 2. Both predictors improved model fit with respect to Model 2 but their direct comparison using Information Criteria demonstrated a better fit for Model 3a (AIC = 12826, BIC = 12855) compared to Model 3b (AIC = 12832, BIC = 12861). We finally tested whether adding Operand order to Model 3a significantly improved the fit (Model 4 vs. Model 3a, LRT: χ²(1) = 0.02, *p* =.88). This comparison revealed no significant improvement, and Model 4 had higher AIC and BIC values.

**Fig. 5:**
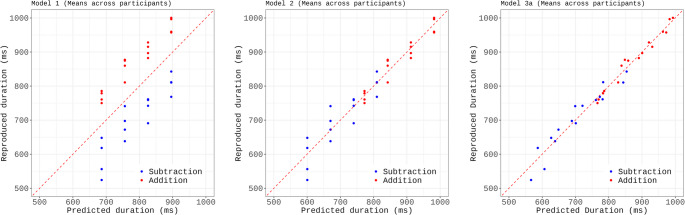
Experiment 2: Scatter plot comparing the observed data (reproduced duration in ms) with the data predicted by the model, which was fit incrementally using Target Duration (model 1, left panel), Operation Type (model 2, middle panel), and Second Operand Duration (model 3, right panel) as predictors. The alignment of the points along the diagonal line is an index of fitness

Therefore, Model 3a, including Target Duration, Operation type, and Second Operand Duration as fixed effects, and a random intercept for subject, was selected as the best-fitting model (see Table 4). Model residuals were normally distributed (Shapiro-Wilk test: W = 0.997, *p* =.22) and variance inflation factors for the fixed effects were low (all VIFs < 1.6), indicating no issues with multicollinearity. The selected model accounted for 57.4% of the variance in the data (conditional R²), with fixed effects explaining 27.6% of the variance (marginal R²). Scatter plots in Fig. 5 contrast the predictions of the nested models with the empirical data for each of the 32 operations, with item means (both observed and predicted) obtained by averaging across participants.

**Table 4:**
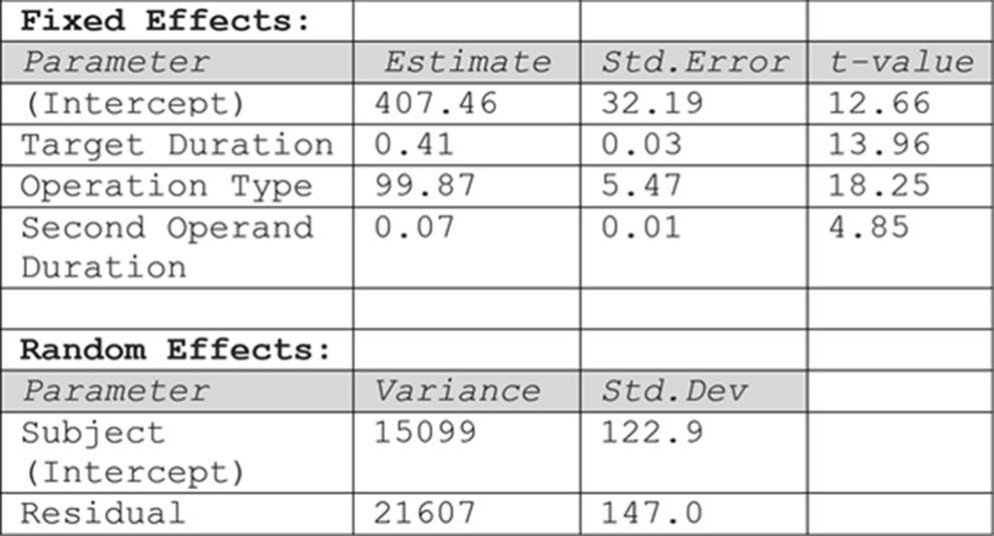
Summary of the best-fitting Linear Mixed-Effects Model predicting reproduced duration in the temporal arithmetic task. The table shows estimates, standard errors, and t-values for fixed effects, and variance and standard deviation for random effects

### Discussion Experiment 2

The results of Experiment 2 confirmed the presence of an arithmetic temporal momentum in a fully balanced set of stimuli (equal number of long first and short first operands) which extended to subtraction trials. For all the temporal durations, the estimates were on average higher for the “Addition” condition compared to the “Subtraction” condition. Furthermore, comparative analyses did not reveal significant differences with Experiment 1 data (characterized by unbalanced subtraction stimuli). Notably, in the “Subtraction” condition the underestimation was present even when only considering the trials in which the second operand was longer than the first. This indicates, for both operations, that the anchoring effect cannot account for the operation-specific pattern of over- and under-estimation, which once more clearly emerged from this second experiment, which extended the findings of Experiment 1 to a set of fully balanced stimuli (subtraction trials included). Finally, regression modeling based on linear mixed models showed that the type of operation (Addition vs. Subtraction) was a crucial factor for predicting the temporal estimates. The duration of the second operand was a better predictor than the order of the operands, and the latter failed to significantly improve model fit when entered in the last step.

## General discussion

Across two online experiments participants were asked to add or subtract the durations of pairs of auditory stimuli (white noise). Their performance was compared with a baseline condition requiring participants to reproduce single white noise stimuli for a duration matching the correct outcome of addition/subtraction. The task did not have any lateralized components as participants were asked to press a response key for reproducing a duration matching the arithmetic outcome. The results confirmed and extended the conditions leading to the temporal momentum effect, an operation-specific bias characterized by longer reproduced durations for addition than for subtraction despite identical target durations. Experiment 1 was a direct replication of an experiment previously performed in the lab. Experiment 2 encompassed a new set of stimuli, including subtractions with shorter first operands. In both experiments, part of the variability in the responses could be explained by a perceptual anchoring effect, whereby participants would adjust their estimates depending on the duration of the second operand. Operands order is known to be a variable which can significantly impact performance (Shaki et al., 2015). On top of this influence, however, regression modeling based on linear mixed models confirmed that our data can satisfactorily be explained only under the assumption of an operation-specific bias.

Experiment 2 involved the use of a fully balanced set of stimulus pairs including shorter first operands not only for addition (as in Experiment 1) but also for subtraction. Accordingly, participants were asked to perform either the sum or the difference between the two operands (and not to subtract the second from the first operand as in Experiment 1). While the anchoring effect was still detected the results confirmed the presence of a significant modulation due to the type of operation being performed. In other words, shorter reproduction in the case of subtraction was still present when subtractions involved an equal number of trials with first operand longer vs. with second operand longer. In the “Subtraction” condition of Experiment 2, the bias was more evident when the second operand was shorter despite the lack of explicit instructions with respect to operands order (i.e., which operand had to be subtracted from which). Linear mixed modeling showed that estimates in the temporal arithmetic task were significantly influenced by the duration of the second operand, which absorbed the putative effect of operand order.

Our findings corroborate those by Bonato et al. (2021) and allow excluding alternative explanations in terms of strategic processes. Broadly speaking, temporal momentum studies significantly expand the literature reporting the classic interactions between magnitudes and response position: the former effect is “conceptual” and it does not hinge upon lateralized responses. The temporal momentum constitutes a “unicum” whereby the pairing between experienced duration and a (putative) spatial component is not directly measured but rather a potential explanation for over-underestimation (i.e., only present at the conceptual level).

The “arithmetic heuristics and biases” (AHAB) model (Shaki et al., 2018; see also Mioni et al., 2021) provides an alternative explanation concerning the origin of the operational momentum by describing a weighted combination of multiple arithmetic heuristics and biases. AHAB suggests that the Operational Momentum in mental arithmetic (as well as the temporal momentum, if we assume a common origin) is due to three different mechanisms whose activation is based on specific conditions:

1. The anchoring bias occurs when result sizes are matched across operation, and it is due to the anchoring to the first operand. Thus, AHAB predicts a reverse Operational Momentum, with larger estimates for subtraction than for addition. It is worth noting that in Shaki et al. (2018) the first operand was always larger than the second.
2. The more-or-less heuristic is due to a common daily-life experience, and it predicts a “classic” Operational Momentum.
3. The sign-space association depends on lateralized responses, and it reflects the associations “addition/right space” and “subtraction/left space”.

As in Mioni et al. (2021), our temporal arithmetic paradigm avoids lateralized responses and consequently the sign-space association cannot account for the findings. Also, the effect was still present after correcting for the impact of the sign (Bonato et al., 2021). Finally, Experiment 2 found identical effects when using a balanced set of stimulus pairs. This leaves only the more-or-less heuristic as a potential account of the results. With these premises, the AHAB model predicts a reverse Operational Momentum for our Experiment 1 (due to predominant presence of the anchoring bias) and a “regular” Operational Momentum for the second experiment (due to the presence of the more-or-less heuristic). The predictions for Experiment 1 were not met. Moreover, our analysis found that people tend to anchor to the second operand (leading to a greater overestimation when the second operand is larger in magnitude) while, in contrast, the AHAB model describes an anchor effect to the first operand (leading to the reverse Operational Momentum). In conclusion, the AHAB model cannot account for our findings, neither fully nor parsimoniously.

A second model to consider is the APiMA model of Prado and Knops (2024). According to it, operand effects depend specifically on their ordering. Specifically, the perceived magnitude of second operand (O2) would be affected by the magnitude of the fist operand (O1). A larger O1 would cause underestimation of O2, whereas a smaller O1 would cause overestimation of O2. This in turn would affect the estimated result (above and beyond the operation effect). However, in the case of Exp. 2, the statistical evidence for an operand order effect was weaker than the evidence for an effect of the second operand when directly compared using information criteria. Moreover, the former was not significant when entered as a predictor in a model that included second operand duration. Thus, while our results can still be interpreted in terms of serial dependencies (Fornaciai & Park, 2020) that affect the arithmetic computation, we conclude that the effect of the second operand is a classic example of anchoring bias (LeBoeuf & Shafir, 2006) rather than the distorted perception of the second operand duration suggested by the APiMA model.

As suggested by one Reviewer we could have been (even) more conservative and avoid presenting the operation sign for each problem, considering that it only varied at the block level. A second, more theoretical criticism to our conclusions could be that they are too general, as for instance “Subtraction” problems do not always produce an underestimation when compared with the “Baseline” condition. For example, in Experiment 1 there was no significant difference in the mean estimates between the two conditions. However, two elements suggest the opposite. First, in Experiment 2 a significant difference between the two conditions emerged. Secondly, the underestimation pattern is visible by taking into consideration the individual temporal durations. The same considerations apply in the few cases in which the “Addition” condition does not seem to lead to a clear overestimation. Note that overestimation for the addition of temporal durations of visual stimuli had been previously observed (e.g., Takahashi & Watanabe, 2015), but no study before Bonato et al. (2021) investigated subtraction of temporal durations. Notably, Takahashi and Watanabe (2015) interpreted the differences found between baseline conditions with no addition and conditions characterized by several additions as an effect of cognitive load. In this respect, a key contribution of the present study is the demonstration that underestimation during subtraction cannot be explained by anchoring or other heuristics. This implies that the overall pattern characterizing the temporal momentum effect is more parsimoniously explained as an outcome of arithmetic processing, and in turn by a putative spatial component in the representation of temporal duration (Bonato et al., 2012). Whether the temporal momentum effect applies to longer durations, such as those characterizing more ecological contexts, is an intriguing question for future investigations.

## Data Availability

All the data of this study are available in a permanent archive accessible at: [https://osf.io/4r2kh/?view_only=516f0334a02541a9a783676cc3023133]; Preregistrations are available at: − Global project [https://osf.io/7hcaf?view_only=516f0334a02541a9a783676cc3023133] − Experiment 1 [https://osf.io/967y3?view_only=516f0334a02541a9a783676cc3023133] − Experiment 2 [https://osf.io/wes78?view_only=516f0334a02541a9a783676cc3023133].

## Declarations

Nothing to disclose.
